# Evidence for a RIPK1-independent survival mechanism for CASPASE-8 in αβ T cells

**DOI:** 10.1093/discim/kyaf016

**Published:** 2025-11-28

**Authors:** Farjana Islam, Scott Layzell, Ines Boal-Carvalho, Benedict Seddon

**Affiliations:** Institute of Immunity and Transplantation, Division of Infection and Immunity, University College London, The Pears Building, Hampstead, London, UK; Institute of Immunity and Transplantation, Division of Infection and Immunity, University College London, The Pears Building, Hampstead, London, UK; Institute of Immunity and Transplantation, Division of Infection and Immunity, University College London, The Pears Building, Hampstead, London, UK; Institute of Immunity and Transplantation, Division of Infection and Immunity, University College London, The Pears Building, Hampstead, London, UK

**Keywords:** apoptosis, t cell, necroptosis, signal transduction

## Abstract

**Introduction:**

CASPASE8 promotes both cell death and survival by acting as a trigger of apoptosis and a repressor of necroptosis. In T cells, the function and mechanisms of CASPASE8 are incompletely understood.

**Methods:**

Here, we analysed mice in which *Casp8* was conditionally deleted in T cells at different stages of development.

**Results:**

In mice with deletion early in T cell development, we observed a modest reduction in early thymic progenitors and a striking absence of NKT cells in the thymus. Amongst mature peripheral T cells, there was a substantial and specific reduction in the CD8 T cell compartment, which included naive, central memory and virtual memory subsets. Using a tamoxifen-inducible CD8^CreERT^ to delete *Casp8* revealed an acute requirement for continued CASPASE8 expression for survival of a fraction of mature CD8 T cells. Analysing *Casp8-*deficient mice that express a kinase dead RIPK1 suggested that *in vivo*, necroptosis contributed to death of thymic progenitors and CD8EM and CD8CM subsets. However, kinase dead RIPK1 failed to restore NKT cell development or rescue the loss of CD4EM and CD4CM in mixed bone marrow chimeras, and only partially rescued CD8 VM T cell.

**Conclusions:**

Together, these observations suggest that CASPASE8 promotes T cell survival independent of its established role in repressing RIPK1-dependent necroptosis.

## Graphical Abstract

**Graphical Abstract:**
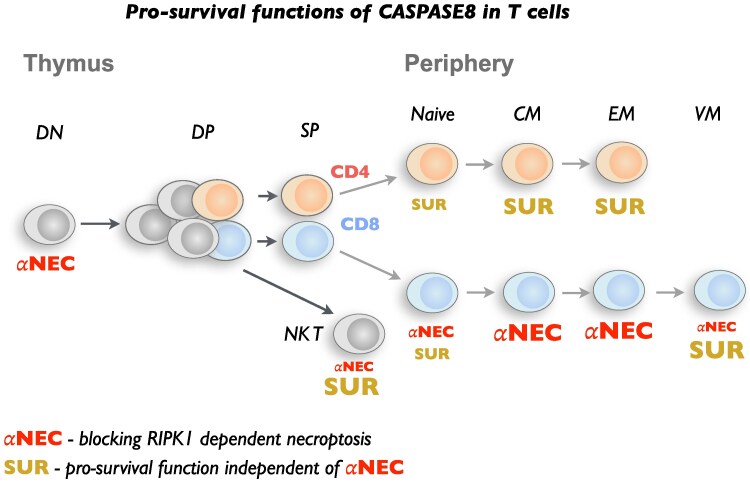

## Introduction

CASPASE-8 is a critical trigger of death downstream of extrinsic cell death pathways. Receptor triggered recruitment of CASPASE8, mediated by the adapter FADD, results in oligomerization of pro-CASPASE8, which leads to proximity-induced autocatalytic cleavage [1]. TNFR superfamily member containing death domains such as Fas (CD95/Apo-1), DR3 (Apo-3), DR4 (Apo-2 or TRAILR1), DR5 (TRAILR2) trimerise upon ligand binding and can directly recruit FADD [2]. In contrast, TNFR1 recruits FADD via the adapter TRADD but only triggers cell death under conditions in which the initial signalling complex-I becomes destabilized [3]. Other receptors capable of recruiting FADD include toll-like receptors [4], NLRP3 and other inflammasomes [5], Dectin-1 [6], and type I interferon receptors [7]. Once cleaved, active CASPASE8 is released into the cytoplasm to cleave and activate executioner caspases (CASPASE3, CASPASE7), resulting in cell death. In some cells, such as hepatocytes and fibroblasts, CASPASE-8 cleaves Bid to tBid, which promotes the release of second mitochondria-derived activator of caspases (SMAC) into cytosol that inhibits IAPs and triggers apoptosis [8, 9]. In addition to inducing cell death, CASPASE-8 also has pro-survival functions since it actively inhibits necroptosis. In the absence of CASPASE8, RIPK1 can recruit RIPK3, which in turn activates the pore forming capacity of MLKL resulting in necroptosis [10]. CASPASE8 is thought to target cleavage of RIPK1, thereby preventing necrosome formation [11, 12]. As such, CASPASE8 is a critical regulator of cell death and cell survival. Mice lacking CASPASE8 die *in utero* at E10.5 as a result of uncontrolled necroptotic cell death, resulting in vascular and haematopoietic defects, which is rescued by additional deletion of RIPK3 [13].

The role of CASPASE8 in T cells has been studied using a variety of conditional gene deletion models, all of which suggest a critical role of CASPASE8 for normal homeostasis of the T cell compartment. However, the precise impact of CASPASE8 on normal maintenance of T cell compartment appears to vary depending on which *Cre* driver was used to mediate conditional gene deletion. Studies employing Lck-Cre report normal thymic development but profound peripheral T cell lymphopenia, even though T cells are resistant to FAS-induced cell death [14]. The same group later report that mice develop a lymphoproliferative disorder that had features resemblant of FAS-deficient *Fas^lpr^* mice, consistent with a loss of CASPASE8-dependent FAS-triggered cell death [15]. Lck-Cre has subsequently been shown to induce significant Cre toxicity [16], which may be a compounding factor in the phenotype exhibited by this strain, which could contribute to, or exacerbate the reduction in peripheral T cell numbers observed in this strain. Studies employing CD4^Cre^, which does not induce such Cre toxicity [16], report normal numbers of thymocytes and peripheral T cells in lymph nodes and spleen [17]. In contrast, subsequent studies suggest a reduction in percentages of T cells in lymph nodes and a reduced frequency of total CD8^+^ T cells, though data did not reach statistical significance [18]. Activation of CASPASE8-deficient T cells, either *in vitro* by CD3 crosslinking or *in vivo* following infection with LCMV, resulted in defective responses [17] that could be rescued either by blocking RIPK1 kinase activity or RIPK3 deletion [18], revealing the role of CASPASE8 in blocking necroptotic cell death in activated T cells. Mice with CD4^Cre^ mediated *Casp8* deletion and with additional loss of RIPK3 resemble *Fas^lpr^* mice, since they developed expansions of CD4^−^CD8^−^ double-negative T cells [18]. While CASPASE8 blocks necroptotic cell death of acutely activated T cells, it is unclear whether resting naive or memory phenotype cells are susceptible to necroptosis in the steady state. CD44^hi^ memory phenotype CD4 and CD8 T cells are reportedly normal in the absence of CASPASE8 [17], and it was not reported whether RIPK3 deficiency rescues the modest reduction in T cell frequencies described in later studies [18]. In contrast, induction of necroptotic cell death amongst Foxp3^+^ regulatory CD4^+^ T cells has also been demonstrated using Foxp3^Cre^-mediated deletion of *Casp8* [19] and results in immune perturbations expected from a loss of regulatory cell activity and that is rescued by additional MLKL ablation.

In the present study, we wished to resolve the apparent discrepancies in the literature around the role of *Casp8* for normal peripheral αβ T cell homeostasis in the steady state and provide a deeper analysis of naive, memory, and unconventional mature αβ T cell subsets. Using huCD2^iCre^ driver line that deletes *Casp8* from early double-negative thymic progenitors and does not induce Cre toxicity [16], we find evidence that CASPASE8 is required for normal thymopoiesis of αβ T cells and in various peripheral mature T cell populations. However, using kinase dead RIPK1 to block necroptotic cell death, we find that only some of these defects resulting from *Casp8* deletion can be accounted for by necroptotic cell death, and that a prosurvival function of CASPASE8 that is distinct from its recognized function in repressing necroptosis, is required to account for the full range of phenotypes.

## Materials and methods

### Mice

Mice with the following mutations were used in this study; conditional alleles of *Casp8*, B6.129-Casp8^tm1Hed^/J (*Casp8^flox^*)*, Cre* transgenes expressed under the control of the human CD2, B6.Cg-Tg(CD2-icre)4Kio/J (*huCD2^iCre^*) [20] mice with a D138N mutation in *Ripk1, B6.129-Ripk1^tm1Geno^/J* (RIPK1^D138N^) [21] and Tg(Cd8a-icre/ERT2, -GFP)Daav (CD8^CreERT2^) [22]. The following strains were bred for this study; *Casp8^fx/fx^ huCD2^iCre^* (Casp8ΔT^CD2^), *Casp8^fx/fx^ huCD2^iCre^* RIPK1^D138N^ (Casp8ΔT^CD2^RIPK1^D138N^), *Casp8^fx/fx^ CD8^CreERT^,* SJL.C57Bl6/J (CD45.1), (SJL.C57Bl6/J × C57Bl6/J)F1 (CD45.1 CD45.2). Both male and female mice were used throughout, taken between ages 8 and 12 weeks.

Cre activity in CD8^CreERT^ mice was induced *in vivo* by i.p. injection of 2 mg of tamoxifen in corn oil for five consecutive days. All mice were bred in the Comparative Biology Unit of the Royal Free UCL campus and at Charles River laboratories, Manston, UK. Animal experiments were performed according to institutional guidelines and Home Office regulations under licence PP2330953.

Mixed bone marrow chimeras were generated by irradiating CD45.1 C57Bl6/J hosts with 800 RADS, followed by injection with total 10^7^ T- and B- depleted bone marrow cells. Bone marrow was isolated from WT CD45.1 CD45.2 C57Bl6/J donors and from Casp8ΔT^CD2^ and Casp8ΔT^CD2^ RIPK1^D138N^ strain mice that are both CD45.2. Bone marrow from CD45.1 CD45.2 donors was mixed 1:1 with either Cre −ve or Cre +ve donors from the different *Casp8* strains.

### Flow cytometry and electronic gating strategies

Flow cytometric analysis was performed with 4–5 × 10^6^ thymocytes, lymph node, or spleen cells. Cell concentrations of thymocytes, lymph node, and spleen cells were determined with a Scharf Instruments Casy Counter. Cells were incubated with saturating concentrations of antibodies in 100 μl of Dulbecco’s phosphate-buffered saline (PBS) containing bovine serum albumin (BSA, 0.1%) for 1 hour at 4°C followed by two washes in PBS-BSA. Panels used the following mAb: PE-Cy7-conjugated antibody against CD25 (eBioscience), PE-conjugated antibody against CD127 (ThermoFisher Scientific), BV785-conjugated CD44 antibody (Biolegend), BV421-conjugated antibody against CD4 (Biolegend), BUV395-conjugated antibody against CD8 (BD Biosciences), BV711-conjugated antibody against CD24 (BD Biosciences), PerCP-Cy5.5-conjugated antibody against TCR (Cambridge Biosciences), FITC-conjugated antibody against CD5 (eBiosciences), BV650-conjugated antibody against CD45.1 (Biolegend), FITC-conjugated antibody against CD45.2 (ThermoFisher Scientific), APC-conjugated antibody against CD49d (Biolegend), FITC-conjugated antibody against Ki67 (ThermoFisher Scientific), PE-conjugated CD1d tetramers (NIH Tetramer facility) complexed with glycolipid α-galactosylceramide (CD1d-α-GalCer). Intracellular staining for Ki67 antigen was performed using Foxp3 fix-perm kit (Biolegend), following manufacturers instructions, to permeabilize and fix samples, followed by anti-Ki67 staining. Cell viability was determined using LIVE/DEAD cell stain kit (Invitrogen Molecular Probes), following the manufacturer’s protocol. Multi-colour flow cytometric staining was analysed on a LSRFortessa (Becton Dickinson) instrument, and data analysis and colour compensations were performed with FlowJo V10 software (TreeStar). Live lymphocytes were gated using standard combination of FSC vs SSC, singlets identified by FSC-H vs FSC-A, and residual dead cells excluded on the basis of live dead discrimination dye (Supplementary Figure S3).

The following gating strategies were used to identify specific subsets of peripheral T cells and thymic populations: peripheral CD4 naive T cells—CD4^+^ TCRβ^+^CD25^−^CD44^lo^CD62L^hi^, CD4 central memory (CM) T cells—CD4^+^ TCRβ^+^CD25^−^CD44^hi^CD62L^hi^, CD4 effector memory (EM) T cells—CD4^+^ TCRβ^+^CD25^−^CD44^hi^CD62L^lo^, CD8 naive T cells—CD8^+^ TCRβ^+^CD44^lo^CD62L^hi^CD49d^lo^, CD8 CM—CD8^+^ TCRβ^+^CD44^hi^CD62L^hi^CD49d^hi^, CD8 virtual memory (VM) T cells—CD8^+^ TCRβ^+^CD44^hi^CD62L^hi^CD49d^lo^ [23] and CD8 EM—CD8^+^ TCRβ^+^CD44^hi^CD62L^lo^. Immature and mature CD4^+^ SP thymocytes were identified as CD4^+^ CD8^−^TCRβ^+^HSA^hi^CD62L^lo^ and CD4^+^ CD8^−^TCRβ^+^HSA^lo^CD62L^hi^, respectively. Immature and mature CD8^+^ SP thymocytes were identified as CD4^−^ CD8^+^TCRβ^+^HSA^hi^CD62L^lo^ and CD4^–^ CD8^+^TCRβ^+^HSA^lo^CD62L^hi^ respectively.

Cell numbers of any given population were calculated by multiplying the total cell recovery of the given organ, determined by CASY counter, by the fraction of the given subset amongst total live lymphocytes.

### *In vitro* culture

Lymph node T cells were cultured at 37°C with 5% CO2 in RPMI-1640 (Gibco, Invitrogen Corporation, CA) supplemented with 10% (v/v) fetal bovine serum (FBS) (Gibco Invitrogen), 0.1% (v/v) 2-mercaptoethanol βME (Sigma-Aldrich) and 1% (v/v) penicillin-streptomycin (Gibco Invitrogen) (RPMI-10). Recombinant TNF was supplemented to cultures at 20 ng/ml, unless otherwise stated, and was obtained from Peprotech, with PBS used as vehicle. Recombinant FASL (Enzo Life Sciences UK Ltd.) was supplemented to cultures at indicated concentrations together with fixed concentration (10 μg/ml) of cross-linking enhancer. Inhibitors were used at the following concentrations, unless otherwise stated: IKK16 (2 µM in 0.1% DMSO).

### Statistics

Statistical analysis, line fitting, regression analysis, and figure preparation were performed using Graphpad Prism 8. Two-way comparison of Cre− and Cre+ column data compared by nonparametric unpaired Mann–Whitney Student’s *t* test. ns = nonsignificant, *P < 0.05, **P < 0.01, *** *P* < 0.001, ****P < 0.0001. Dose–response curves 2-way ANOVA, life spans by exponential decay curve fits with 95% confidence intervals.

## Results

### CASPASE8 expression is required for optimal expansion of DP thymic compartment but not selection or development of SPs

**Figure 1.**
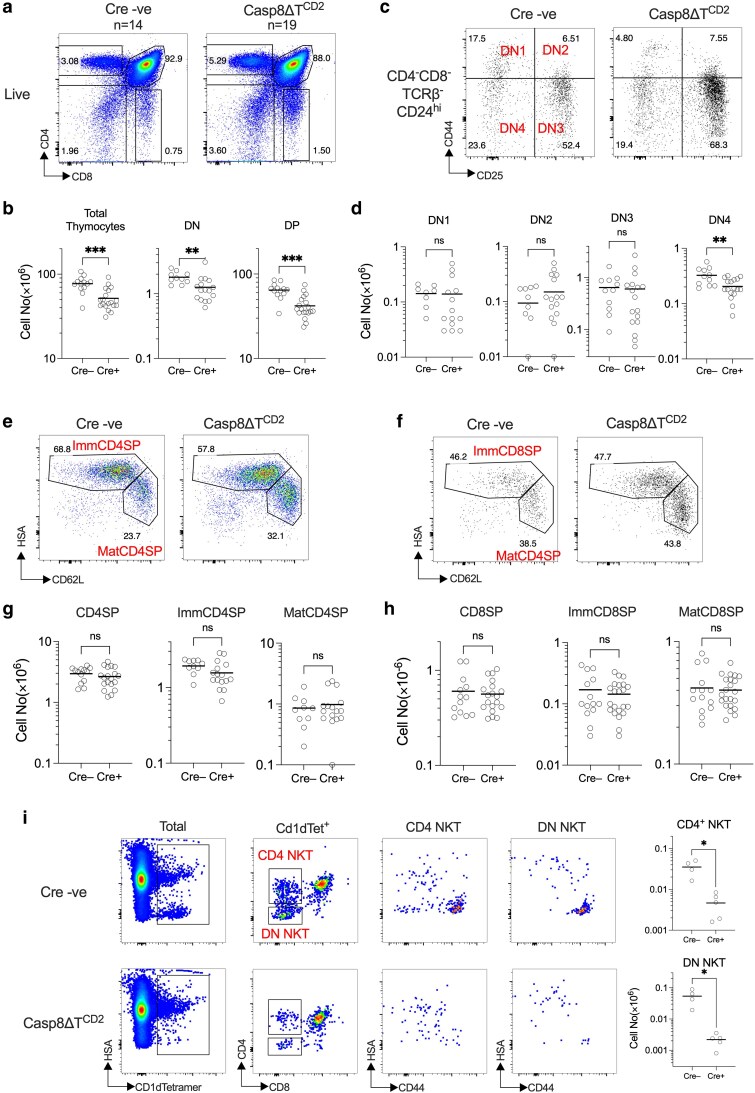
CASPASE8 deficiency impacts **α**β T cell development. Thymi from Casp8**Δ**T^CD2^ mice (*n* = 19) (Cre+ on scatter plots) and Cre −ve littermates (Cre− on scatter plots) (*n* = 14) were enumerated and analysed by flow. (a) Density plots are representative expression of CD4 vs CD8 by live gated thymocytes. (b) Scatter plots show the numbers of total thymocytes, CD4^−^CD8^−^ double-negative (DN) thymocytes and CD4^+^CD8^+^ double-positive (DP) thymocytes. (c) Dot plots are of CD44 vs CD25 expression by CD4^−^CD8^−^TCRβ^−^ HSA^hi^ thymocytes from representative mice. Quadrants used to identify DN1, DN2, DN3, and DN4 subsets are labelled on the plot of control mouse. (d) Scatter plots summarize numbers of the indicated DN subset. (e and f) Density and dot plots of HSA vs CD62L are from CD4^+^CD8^−^ TCRβ^+^ (e) and CD4^−^CD8^+^ TCRβ^+^ (f) live thymocytes. Labels indicate the gates used to identify immature (Imm) CD62L^lo^HSA^hi^ and mature (Mat) CD62L^lo^HSA^hi^ subsets. (g and h) Scatter plots summarize the total numbers of total SP, immature HSA^hi^CD62L^lo^, and mature HSA^lo^CD62L^hi^ SP subsets for CD4 (g) and CD8 (h) lineages. (i) Density plots are of HSA vs CD1d-α-GalCer (CD1dTet) binding to total live thymocytes. CD4 vs CD8 expression is for CD1dTet^+^ cells. Gates used to identify CD4^+^CD8^−^ CD1dTet^+^ (CD4 NKT) and CD4^−^CD8^−^ CD1dTet^+^ (DN NKT) cells are labelled on the plots. HSA vs CD44 expression is shown for CD4 NKT cells and DN NKT cells. Scatter plots summarize total numbers of CD4 NKT and DN NKT recovered from mice (*n* = 4 Cre− and *n* = 4 Cre+ mice). Data are pooled from multiple batches of mice analysed.

To assess the impact of *Casp8* deletion on thymic development, we generated *Casp8^fx/fx^* huCD2^iCre^ mice (Casp8ΔT^CD2^) in which conditional *Casp8* alleles are deleted by an iCre expressed from a huCD2 regulated transgene [20]. huCD2^iCre^ mediates gene deletion in T and B cells. In the T cell lineage, it is first expressed in the thymus, in immature CD4^−^CD8^−^ double-negative (DN) thymocytes. There are four further subsets of DN thymocytes, DN1 to DN4, defined by expression of different combinations of CD44 and CD25. huCD2iCre activity is first detected amongst a subset of DN1 and DN2 thymocytes, but active in all thymocytes by the DN3 stage. We first analysed the thymi from *Casp8^fx/fx^* huCD2^iCre^ mice (Casp8ΔT^CD2^) as compared with Cre −ve *Casp8^fx/fx^* littermates. The distribution of subsets defined by CD4 and CD8 expression was largely normal. A modest but significant reduction of total thymus size was evident in the absence of CASPASE8, which was reflected in a reduction in size of both CD4^−^CD8^−^ double-negative (DN) and CD4^+^CD8^+^ double-positive (DP) compartments (Fig. 1a and b). Analysing the size of precursor DN1-4 subsets revealed normal DN1-3 numbers but a reduced DN4 compartment (Fig. 1c and d). The interpretation of DN1 and DN2 phenotype was tempered by the fact that huCD2^iCre^ is not expressed in these subsets with a maximal penetrance [16, 20]. In contrast, this iCre is expressed in >99% of DN3 and DN4 subsets. Other aspects of conventional T cell development appeared normal. CD4 and CD8 single positive (SP) compartments were largely normal in size and maturation within these compartments also appeared normal (Fig. 1e–h), suggesting the loss in DP cellularity was not sufficient to adversely impact the size of mature post-selection compartments and therefore thymic output. However, analysing the development of NKT cells, identified by their binding to CD1d tetramers complexed with glycolipid α-galactosylceramide (CD1d-α-GalCer), revealed a profound defect in NKT cells within the thymus. This appeared to be associated with a failure of mature CD44^hi^ NKT cells to accumulate, since CD1d-α-GalCer binding precursors amongst DP thymocytes appeared normal as were immature HSA^hi^ precursors amongst DN and CD4 SP populations [24] (Fig. 1i).

### Perturbations of peripheral CD8 T cell subsets in the absence of CASPASE8 expression

**Figure 2.**
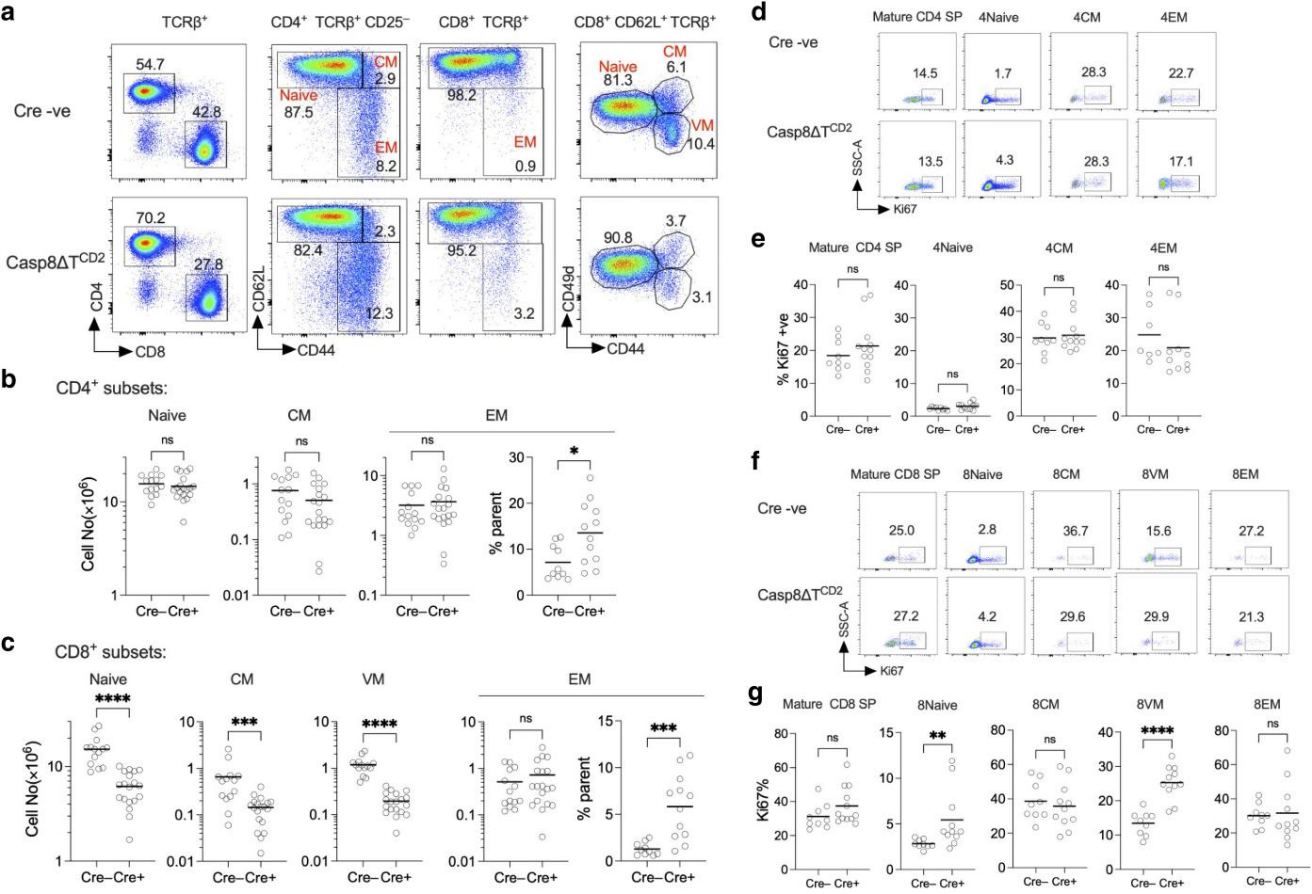
Reduced peripheral CD8 compartment size following *Casp8* deletion. Lymph nodes and spleen from the cohorts of mice described in Fig. 1 were enumerated and analysed by flow cytometry. (a) Density plots are of CD4 vs CD8 by TCRβ^+^ lymph node cells, CD62L vs CD44 by either CD4^+^ TCRβ^+^ CD25^−^ or CD8^+^ TCRβ^+^ subsets. Gates used to identify CD4^+^ naive, central memory (CM) and effector memory (EM) subsets are labelled in red. Naive, CM and virtual memory (VM) CD8 subsets were identified amongst CD8^+^ CD62L^hi^ TCRβ^+^ cells using CD49d and CD44 as indicated. (b and c) Scatter plots summarized the total numbers of the indicated CD4 (b) and CD8 (c) subsets from total lymph nodes and spleen combined from Cre− (*n* = 14) and Cre + (*n* = 19) *Casp8^fx/fx^* mice. (d–g) Density plots are of SSC vs Ki67 by the indicated subsets of CD4 (d) and CD8 (f) T cell subsets in thymus and lymph nodes. Scatter plots (e and g) summarize the frequency of Ki67^+^ cells amongst the indicated subsets from Cre− (*n* = 9) and Cre+ (*n* = 11) *Casp8^fx/fx^* mice.

We next analysed the size and detailed composition of peripheral compartments of CD4 and CD8 T cells in lymph nodes and spleen, to determine the impact of *Casp8* deletion. Analysing the CD4 T cell compartment revealed normal size and composition, with the exception that CD4 effector memory (EM) compartment appeared slightly enlarged, relative to other subsets (Fig. 2a). This was not, however, reflected in a detectable change in absolute cell numbers recovered from mice (Fig. 2b). In contrast, the CD8 compartment exhibited alterations in all subsets as compared with Cre− controls. Numbers of naive CD44^lo^, CD62L^hi^ CD49d^hi^ CD44^hi^ central memory (CM), and CD62L^hi^ CD49d^lo^ CD44^hi^ virtual memory (VM) subsets were all reduced (Fig. 2c). In contrast, absolute numbers of CD62L^lo^ CD44^hi^ effector memory (EM) CD8 cells were similar to controls, in spite of the reduction in numbers of other subsets, suggesting a relative increase in EM subset, and this was reflected by an increased representation of these cells amongst total CD8^+^ T cells in Casp8ΔT^CD2^ mice (Fig. 2c).

Alterations in the size of different CD8 subpopulations could result from several causes, including reduced self-renewal capacity of populations that would be evidenced by reduced cell division. To assess altered cell division activity as a potential cause of the defects observed in the CD8 compartment, we assessed expression of the nuclear antigen Ki67 that is expressed by cells during mitosis, and for a short period after. We assessed Ki67 expression amongst SP subsets in the thymus, which undergo limited division before egress [25], and by naive, CM, EM, and VM subsets in the periphery (Fig. 2d–g). In general, we observed no reduction in Ki67 expression that might reflect reduced self-renewal processes that could account for reductions in T cell populations. Ki67 expression was, in fact, increased by CD8 naive and CD8 VM T cells (Fig. 2f, g). Most Ki67 expression amongst naive T cells of mice derives from expression by recent thymic emigrant that have undergone cell division in the thymus [25]. The small increase amongst CD8 naive cells in Casp8ΔT^CD2^ mice may therefore reflect normal thymic output into a reduced peripheral CD8 T cell compartment. In the case of CD8 VM T cells, this compartment is greatly reduced in the absence of CASPASE8. CD8 VM turnover is driven by IL-15 [26], so the increased cell division in the remaining CD8 VM cells in Casp8ΔT^CD2^ mice may reflect increased homeostatic division in response to a substantially emptied niche. Nevertheless, we found no evidence that the reduction in CD8 subsets could be simply explained by a failure of self-renewing cell division processes.

### Normal survival signalling and dynamics in the absence of CASPASE8

**Figure 3.**
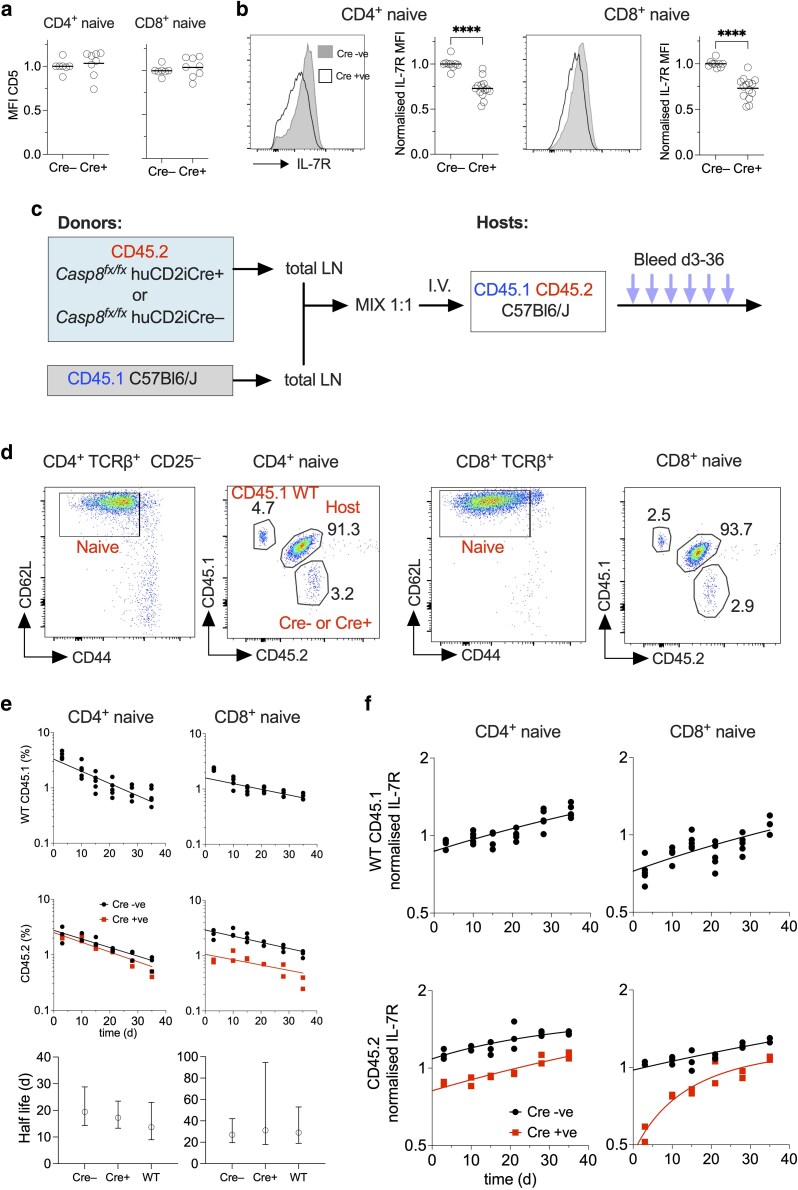
Normal survival signalling and dynamics of T cells from Casp8ΔT^CD2^ donors. (a) Scatter plots are of MFI of CD5 expression normalized to the average level in Cre −ve T cells of the indicated naive subsets from either Casp8ΔT^CD2^ (Cre+) or littermate Cre− controls. (b) Histograms are of IL-7R expression by naive CD4 and CD8 T cells from the indicated donors, and scatter plots are a summary of expression normalized to average MFI of Cre −ve controls. (c) Experimental design for adoptive transfer of CASPASE8-deficient T cells into WT hosts. (d) Gating strategy to track the indicated Cre+ (*n* = 2), Cre− (*n* = 3) or WT CD45.1 (*n* = 5) donor populations in CD45.1^+^CD45.2^+^ hosts amongst naive CD4 and naive CD8 populations in blood. (e) Scatter plots summarize representation of donor populations amongst total naive CD4 and naive CD8 populations in blood. Lines are best fit estimates of exponential decay of the corresponding populations. Bottom plots show that half lives and 95% confidence intervals derived from these fits to the indicated donor populations. (f) Scatter plots show expression of IL-7R, normalized to average MFI of host subsets. Data are representative of two independent experiments.

We next assessed homeostatic survival signalling in naive T cells in Casp8ΔT^CD2^ mice. TCR-induced survival signals are required for optimal naive T cell survival and can be assessed by measuring the surrogate marker CD5 [27]. CD5 expression appeared at normal levels in both CD4 and CD8 naive T cells from Casp8ΔT^CD2^ mice implying similar extents of TCR signalling (Fig. 3a). IL-7 is also an essential survival cue for naive T cells, and IL-7 signalling is regulated in T cells at the level of IL-7R expression [28]. Measuring IL-7R levels by naive T cells did reveal a significant modest reduction in the levels of IL-7R protein expressed by both CD4 and CD8 naive T cells (Fig. 3b).

The reduction in IL-7R could potentially result in reduced life span of naive T cells that would account for altered compartment sizes. We noted, however, that CD4 naive T cell numbers appeared normal in Casp8ΔT^CD2^ mice, in spite of similarly reduced IL-7R expression. Nevertheless, we sought to assess survival of naive T cells from Casp8ΔT^CD2^ mice to determine whether a population wide survival defect could account for the reduction in the CD8 T cell compartment. To do this, we compared the lifespan of CASPASE8 deficient CD4 and CD8 T cells with those of WT subsets following their adoptive transfer into normal, WT hosts (Fig. 3c). We isolated total T cells from Cre +ve and Cre −ve littermates and transferred them into congenic CD45.1^+^CD45.2^+^ WT hosts. As further control, we also co-transferred WT T cells from CD45.1 donors alongside either Cre −ve and Cre +ve donor cells. Hosts were periodically bled and representation of donors T cell subsets assessed (Fig. 3d), together with IL-7R expression level. As expected, control CD45.1 and Cre −ve donor CD4 and CD8 naive T cells steadily declined in number over time for both subsets (Fig. 3e), in line with previous studies and expected life spans [29]. Analysing survival of T cells from Casp8ΔT^CD2^ donors revealed near identical profiles of loss as exhibited by control T cells (Fig. 3e). Representation of CD8^+^ naive T cells from Casp8ΔT^CD2^ donors started from a lower point since they were reduced in numbers in the original donors. However, their decay thereafter tracked that of control populations, and there were no significant differences in estimated half lives of any of the populations assessed (Fig. 3e).

Analysing IL-7R expression showed that the reduced expression level amongst Casp8ΔT^CD2^ donor T cells was preserved following transfer (Fig. 3f). However, in all cases, the measured level of IL-7R on surviving T cells gradually increased over time. Therefore, we found no evidence that the lifespan of naive CD8 T cells from Casp8ΔT^CD2^ mice was reduced compared with controls, and the small difference in IL-7R expression we observed did not appear to impact life span.

### Continued CASPASE8 expression is essential for naive T cell survival

Our adoptive transfer experiments suggested that life span of T cells from Casp8ΔT^CD2^ mice was normal compared to that of WT T cells. However, the cells we assessed were those that persist into adulthood in Casp8ΔT^CD2^ mice and may represent a subpopulation that had been subject to counter selection over the life of the mouse, thus enriching for longer-lived cells at the expense of shorter-lived ones. This would reconcile the observations of normal lifespans but reduced compartment size, specifically for CD8 T cells. If true, this would predict that a substantial fraction of the normal peripheral CD8 T cell compartment should be reliant on continued CASPASE8 expression for their normal survival. To test this, we bred *Casp8^fx^* mice with CD8^CreERT^ transgenic mice that express a tamoxifen-inducible CreERT2 construct under control of the CD8 E8i promoter (CD8^CreERT^ hereon). In these mice, E8i expression elements switch on CreERT expression late in thymic development, first appearing in a small subset of mature CD62L^hi^HSA^lo^ CD8SP thymocytes but not DP thymocytes or immature CD8SP. Expression is maintained throughout the peripheral CD8 compartment thereafter [22]. As such, this Cre driver only targets post-selection CD8 T cells and following Cre-mediated gene deletion, *de novo* generation of CD8 T cells from the thymus would be anticipated to replenish the peripheral compartment with WT CD8 T cells.

It has not previously been reported whether CD8^CreERT^ driver mediates Cre toxicity. Therefore, we first tested the impact of our tamoxifen treatment regime, of five daily injections, into hemizygous CD8^CreERT^ mice. We quantified CD8 T cell numbers at d7, to assess acute Cre toxicity, and also at d21, which is a time point we would assay the impact of induced *Casp8* deletion. This timing was informed by previous studies of CD4 T cell survival, following tamoxifen-inducible CreER-mediated deletion of survival genes [30], and studies that show that only 10% of the CD8 T cell compartment is replenished by *de novo* thymic output over a 3-week period in young adult mice [31]. Analysing tamoxifen-treated hemizygous CD8^CreERT^ mice shows that numbers of CD8 naive and CD8 CM T cells were normal at d7 and d21 after first tamoxifen injection. CD8 EMs were reduced by around half while CD8 VM were reduced by a third, suggesting that there was some limited *Cre* toxicity in these subsets (Supplementary Figure S1). These changes were already evident by Day 7 and remained stable thereafter until Day 21, strongly suggesting that acute Cre toxicity was responsible for these perturbations to VM and EM subsets.

**Figure 4.**
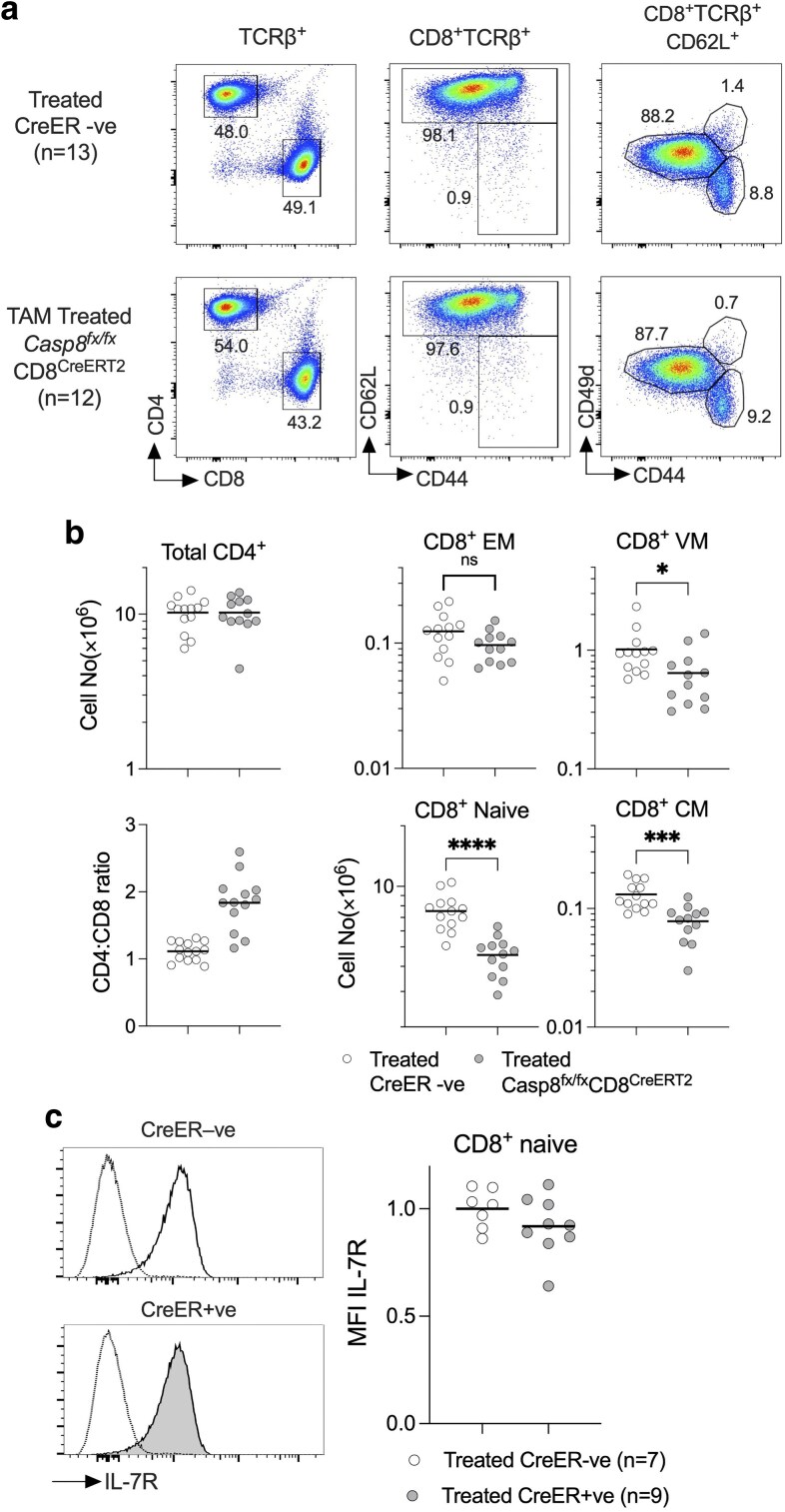
CD8 T cells require constitutive CASPASE8 expression for their survival. *Casp8^fx/fx^* CD8^CreERT^ mice were treated with TAM for five consecutive days. At Day 21 after first injection, mice were culled and CD8 T cell compartment in lymph node and spleen enumerated. (a) Density plots show representative phenotypes in lymph nodes from TAM treated *Casp8^fx/fx^* CD8^CreERT^ mice (*n* = 13) and Cre −ve littermate controls (*n* = 12) and gates used to identify naive, EM, CM, and VM subsets of CD8 T cells. (b) Scatter plots show total numbers of total CD4^+^ TCRβ^+^ T cells, the CD4:CD8 ratio amongst lymph node cells, and total numbers of the indicated CD8 subsets recovered from lymph node and spleen of the indicated strains. (c) Histograms are of IL-7R expression by naive CD8 T cells (filled) vs B cells as negative control, from the indicated strains. Data are pool of four independent experiments. EM, effector memory; CM, central memory; VM, virtual memory.

Since naive and CM subsets were not susceptible to *Cre* toxicity in this strain, we assessed the impact upon these subsets of *Casp8* deletion in *Casp8^fx/fx^* CD8^CreERT^ mice given five injections of TAM on consecutive days. At d21 after the first injection, the T cell compartments of treated mice were analysed. As expected, total CD4^+^ T cell numbers were unaffected following TAM treatment (Fig. 4b). Measuring the CD4:CD8 ratio revealed an increase specifically in TAM-treated *Casp8^fx/fx^* CD8^CreERT^ mice, suggesting that the CD8 T cell compartment was specifically perturbed following CASPASE8 ablation. Analysing specific CD8^+^ subsets revealed that both CD8 naive and CD8 CM numbers were reduced by around half in TAM-treated *Casp8^fx/fx^* CD8^CreERT^ mice (Fig. 4a and b). EM and VM populations were also slightly reduced but only to extents similar to the reductions observed in TAM-treated CD8^CreERT^ single transgenic mice as a result on Cre toxicity (Supplementary Figure S1b). As such, we could not determine whether *Casp8* deletion in these subsets resulted in cell loss. While we observed a reduction of IL-7R expression by CD8 T cells from Casp8ΔT^CD2^ mice, following inducible *Casp8* deletion, IL-7R levels did not appear to be altered on CD8 T cells (Fig. 4c).

### CASPASE8-deficient T cells are resistant to both TNF- and FAS-induced cell death *in vitro*

The loss of naive T cells following deletion of *Casp8* suggested that CASPASE8-deficient T cells were susceptible to cell death by some mechanism. The proteolytic activity of CASPASE8 is known to repress necroptotic cell death by targeting RIPK1 [11 ] and thereby prevent formation of the necroptosome. In T cells, this activity of CASPASE8 has already been described in activated T cells but not resting subsets of naive or memory T cells, which are not thought to express MLKL. Nevertheless, we wished to test whether necroptotic cell death in the absence of CASPASE8 expression could account for the loss of peripheral naive CD8 T cells.

**Figure 5.**
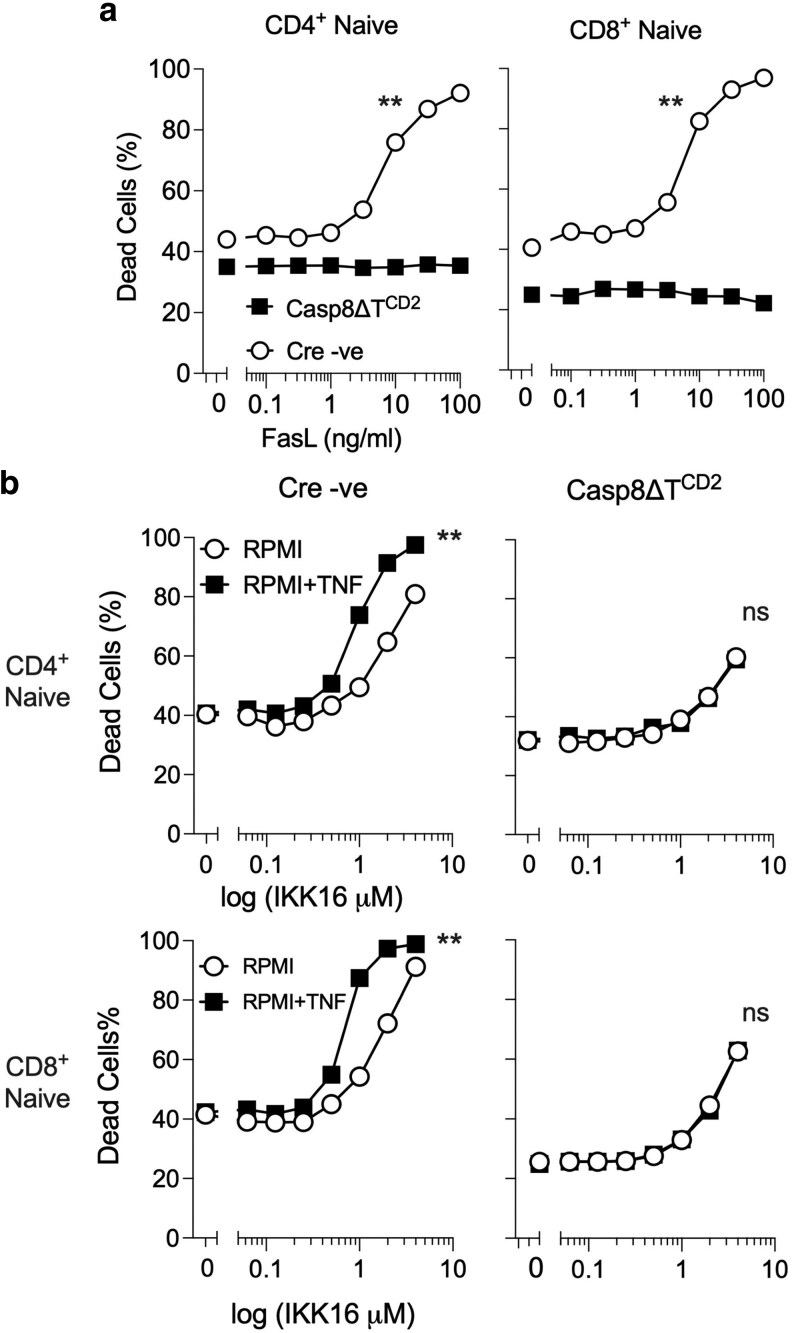
CASPASE8-deficient T cells are resistant to TNFRSF induced death. (a) T cells from Casp8ΔT^CD2^ mice or Cre −ve littermates were cultured overnight with a titration of FASL and crosslinking reagent, and then cell viability of naive CD4^+^ and CD8^+^ T cells assessed using a Live/Dead dye by flow cytometry. (b) T cells were cultured with a titration of pan-IKK inhibitor, IKK16, in the presence and absence of TNF. Graphs show cell death of the indicated naive T cells subsets (rows) from the indicated mouse genotype (columns) with increasing IKK16 concentrations in the presence or absence of TNF. Data are representative of three independent experiments. Cell death responses were compared by 2-way ANOVA. **<0.01. ns, not significant.

First, we tested whether CASPASE8-deficient T cells were sensitive to death induced by TNFRSF members. We challenged CASPASE8-deficient T cells with two death inducing stimuli—(i) FAS stimulation with recombinant FLAG tagged FASL protein and crosslinked with anti-FLAG monoclonal antibody and (ii) TNF in the presence of panIKK inhibitor (IKK16). In the latter case, IKK inhibition sensitizes T cells to RIPK1-dependent cell death when stimulated with TNF [30]. At high doses, IKK16 inhibitor is cytotoxic in a RIPK1-independent manner and likely reflects off target effects of the inhibitor. Therefore, we cultured T cells in a range of IKK16 concentrations and assessed cell viability in the presence and absence of TNF. In the absence of CASPASE8 expression, both naive CD4 and naive CD8 T cells were resistant to FASL-induced cell death observed in WT T cells from control mice (Fig. 5a). Similarly, while T cells from control mice were sensitized to TNF-induced cell death in the presence of the IKK inhibitor IKK16, *Casp8* deficient T cells were completely resistant to TNF-dependent cell death (Fig. 5b). In conclusion, the naive T cells present in Casp8ΔT^CD2^ mice appear resistant to both cell death inducing stimuli with no evidence of either apoptotic or necroptotic cell death following *in vitro* challenge with ligands.

Of incidental note, we have previously shown that cytotoxicity of IKK16 inhibitor at high doses, in the absence of TNF, is RIPK1 independent [30]. Here, our results suggest that cytotoxicity is also CASPASE8 independent, since similar profile of cell death was observed in the absence of CASPASE8 expression. Thus, the cytotoxic effects of IKK16 appear to be off target effects that are independent of triggering CASPASE8-dependent extrinsic cell death pathways.

### CASPASE8-dependent survival of T cells is only in part dependent upon RIPK1 kinase activity

Culturing T cells from Casp8ΔT^CD2^ mice with TNF and FAS ligand *in vitro* did not reveal any evidence that CASPASE8-deficient T cells were sensitized to any form of cell death in the absence of CASPASE8 that might account for loss of T cells *in vivo*. Furthermore, T cells from Casp8ΔT^CD2^ mice appear to survive normally *in vivo*, since they exhibited normal lifespans upon transfer to WT mice. However, induced deletion of *Casp8* in normal peripheral CD8 T cells did result in a rapid loss of a fraction of the peripheral CD8 compartment. To reconcile these observations, it is possible that those T cells that persist in adult Casp8ΔT^CD2^ mice are resistant to cell death and may not be fully representative of a normal replete WT CD8 T cell compartment.

**Figure 6.**
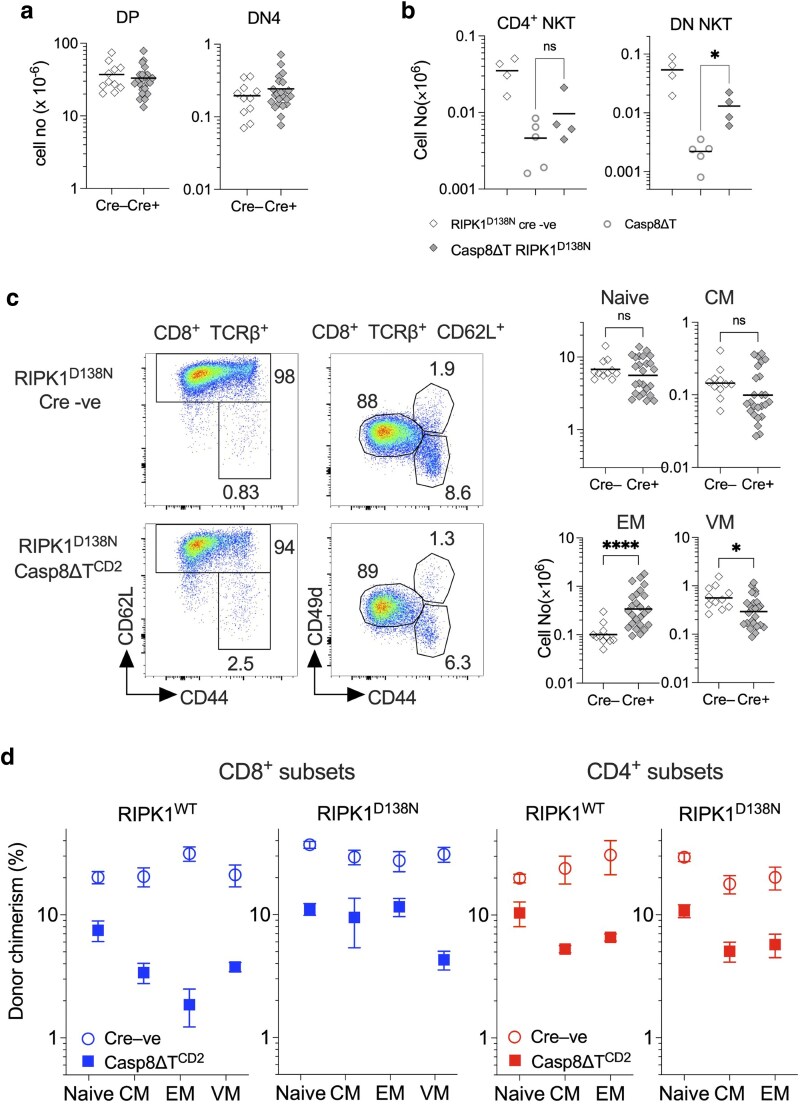
Partial rescue of T cell deficiencies in *Casp8*-deficient mice by kinase dead RIPK1. Lymphoid organs (thymus, LN, and spleen) from Casp8ΔT^CD2^ RIPK1^D138N^ (*n* = 25) and Cre −ve littermates (*n* = 11) were enumerated analysed by flow. (a) Scatter plots show numbers of CD4^+^CD8^+^ DP and DN4 subsets in thymus of the indicated mice. (b) Scatter plots are of numbers of CD1d-α-GalCer binding CD4^+^ and DN NKT cells in thymus of the indicated strains. (c) Density plots are of CD62L vs CD44 by TCRβ^+^CD8^+^ lymph node cells and show gates used to identify CD8 EM and CD62L^hi^ CD8^+^TCRβ^+^ T cells. Density plots of CD49d vs CD44 by CD62L^hi^ CD8^+^TCRβ^+^ T cell show gates used to identify naive, CM and VM subsets. Scatter plots are of total numbers of the indicated subsets of CD8^+^ T cells recovered from both spleen and lymph nodes of the indicated strains. (d) Mixed bone marrow chimeras were generated by reconstituting CD45.1 hosts (*n* = 4 per condition) with mixture of bone marrow from CD45.2 CD45.1 WT congenic donors and bone marrow from Cre + or Cre −ve donors of either Casp8ΔT^CD2^ or Casp8ΔT^CD2^ RIPK1^D138N^ donors (both CD45.2) (see Materials and Methods). Eight weeks later, the composition of CD45.2 donor compartments was measured. Dot plots show the contribution of *Casp8*^WT^- and *Casp8*-deficient cells to the indicated subsets of CD4 and CD8 T cells, originating from either RIPK1^WT^ donors (top) or RIPK1^D138N^ expressing donors, as a fraction of the total donor CD45.2 compartment. Data are representative of three independent experiments.

Since CASPASE8 has a well characterized role in repressing necroptosis, we wished to directly test, genetically, whether necroptotic cell death could account for any of the phenotypes we observed in Casp8ΔT^CD2^ mice. We did this by breeding and analysing Casp8ΔT^CD2^ mice expressing a kinase dead RIPK1^D138N^ mutant [21]. RIPK1 is a critical adaptor, triggered by its autophosphorylation, to promote formation of the necroptosome and induce necroptosis following TNFRSF signalling [10]. Therefore, kinase dead RIPK1 is anticipated to block any necroptotic cell death processes in T cells in Casp8ΔT^CD2^ mice. Our prior analysis shows that loss of RIPK1 kinase activity does not impact thymic development [32]. We first analysed the thymus of Casp8ΔT^CD2^ RIPK1^D138N^ mice to see whether the reduced thymus size, driven by reductions in DN4 and DP populations, and the absence of mature NKT cells in Casp8ΔT^CD2^ mice was dependent upon RIPK1 kinase activity. Comparing Cre –ve and Cre +ve littermates from Casp8ΔT^CD2^ RIPK1^D138N^ mice showed that numbers of DN4 and DP thymic subsets were, in both cases, similar between Cre −ve and Cre +ve mice (Fig. 6a). This was also true for all the other major thymic subsets that were also unaffected in Casp8ΔT^CD2^ mice (Supplementary Figure S2a). In contrast, mature NKT cell numbers in the thymus of Casp8ΔT^CD2^ RIPK1^D138N^ mice displayed a very limited but detectable rescue when RIPK1 kinase was inactive (Fig. 6b and Supplementary Figure S2b). Therefore, we did find evidence of RIPK1 dependent necroptosis amongst thymocytes following CASPASE8 ablation that modulated thymic development.

CD4^+^ T cell numbers were normal in Casp8ΔT^CD2^ mice, so we next focused attention on the peripheral CD8 compartment in Casp8ΔT^CD2^ RIPK1^D138N^ mice. In Casp8ΔT^CD2^ mice, there were significant reductions in naive, CM and VM CD8^+^ subsets and a non-significant increase in EM (Fig. 2). In Casp8ΔT^CD2^ RIPK1^D138N^ mice, numbers of naive and CM subsets were similar between Cre −ve and Cre +ve littermates, while the VM compartment was only reduced to around half that of Cre −ve control mice, suggesting RIPK1^D138N^-mediated partial rescue of this population (Fig. 6c). The CD8 EM compartment was more robustly expanded in Casp8ΔT^CD2^ RIPK1^D138N^ mice (Fig. 6c) than observed in Casp8ΔT^CD2^ mice (Fig. 2).

Analysis of intact Casp8ΔT^CD2^ RIPK1^D138N^ mice provided evidence that necroptosis did account for some but not all of the phenotypes observed in Casp8ΔT^CD2^ mice. However, because the mutant RIPK1^D138N^ is expressed in all cells of these hosts, we wished to confirm the cell intrinsic nature of the rescue by generating mixed bone marrow chimeras using normal hosts and WT partner bone marrow progenitors alongside experimental bone marrow. Irradiated CD45.1 hosts were reconstituted with a mixture of control bone marrow from CD45.1 CD45.2 WT donors and bone marrow from either Cre + or Cre −ve donors from Casp8ΔT^CD2^ strain mice or Casp8ΔT^CD2^ RIPK1^D138N^ mice. Hosts were allowed to reconstitute for 8 weeks and then the donor composition of CD4 and CD8 T cell compartments analysed across naive, CM, EM, and VM subsets. As expected, donor reconstitution, relative to control WT CD45.1 CD45.2 WT competitor, by bone marrow from WT Cre −ve littermates of Casp8ΔT^CD2^ mice exhibited a consistent and stable contribution across different subsets of both CD4 and CD8 T cells (Fig. 6d). In contrast, reconstitution of CD8 compartments by bone marrow from Casp8ΔT^CD2^ mice was poor, with a low level of competitive chimerism observed amongst naive CD8 T cells that was progressively reduced in CM, VM, and EM subsets. Analysing chimerism in CD4 subsets revealed a similar pattern of reconstitution. Representation of *Casp8* deficient T cells was reduced across all CD4 subsets analysed. We compared this pattern of reconstitution with that of chimeras using donor bone marrow from RIPK1^D138N^ mice. Cre −ve bone marrow from RIPK1^D138N^ strains exhibited a consistent and stable contribution across of different subsets of both CD4 and CD8 T cells, similar to that of chimeras using bone marrow from RIPK1^WT^ Cre −ve controls. Amongst CD8 subsets, kinase dead RIPK1^D138N^ did not improve the competitive fitness of *Casp8* deficient naive or VM T cells, which were under represented in much the same manner as in chimeras using bone marrow from Casp8ΔT^CD2^ mice. However, donor representation amongst CM and EM subsets remained stable, suggesting RIPK1^D138N^ did rescue survival of these subsets in the absence of CASPASE8 (Fig. 6d). In contrast, amongst CD4 subsets, kinase dead RIPK1 failed to restore representation of naive, CM and EM subsets, suggesting that necroptosis was not responsible for their loss in the absence of CASPASE8. Together, these data reveal that competitive fitness of T cells is reduced in the absence of CASPASE8 expression, for both CD8 and CD4 subsets, and that necroptosis can only account for the loss of CD8 EM and CD8 CM.

## Discussion

In the present study, we wished to better understand the impact and mechanisms by which CASPASE8 signalling influences the development and maintenance of the T cell compartments of mice. Analysing the chief developmental and mature compartments revealed important survival functions for CASPASE8 at multiple stages of differentiation and in different lineages of T cells. Analysing mice expressing kinase dead RIPK1 revealed that only some of the observed defects of CASPASE8-deficient T cells could be accounted for by a loss of cell death control and induction of RIPK1-dependent necroptosis. Since blocking necroptosis by kinase dead RIPK1 only partially restores normal T cell compartments in Casp8ΔT^CD2^ mice, we can only reconcile the range and complexity of phenotypes we observed, by evoking a novel pro-survival mechanism mediated by CASPASE8 that is independent of its function repressing necroptosis. The composition of T cell compartments in Casp8ΔT^CD2^ mice is therefore the result of a complex, compound phenotype.

While it is clear that activated T cells require CASPASE8 to protect them from induction of necroptosis [17, 18, 33], previous studies have been somewhat equivocal regarding the requirement for CASPASE8 to establish and/or maintain normal homeostasis of mature peripheral T cell compartments [14, 17, 18]. Our detailed analysis revealed defects amongst DN thymocytes, CD8 CM, VM, and NKT cells that had not previously been recognized. In regard to naive T cells, however, evidence appears somewhat conflicting, with reports of both normal compartment size [17] and either selective [18] or broad T cell lymphopenia [14]. In our study, Casp8ΔT^CD2^ mice exhibited a clear reduction in the size of the naive CD8 T cell compartment while numbers of naive CD4 T cells appeared normal. Although we found evidence for a modest impact on thymic development, which may be due to RIPK1-dependent necroptosis amongst DN4 progenitors, numbers of post-selection SP thymocytes were largely normal. It therefore appears that the modest reduction in DP cellularity may be compensated by more efficient positive selection to maintain normal SP compartments and thymic output, and it does not appear that a deficiency in post-selection thymic precursors can account for the substantial reduction in the CD8 T cell compartment.

Amongst peripheral T cells, life spans and turnover of CASPASE8-deficient T cells from Casp8ΔT^CD2^ mice also appeared normal, so we could not account for the reduction in the CD8 T cell compartment by generalized alterations in homeostatic properties of the constituent CD8 T cells. We did find evidence that a subset of naive CD8 T cells may be specifically and acutely dependent on continued CASPASE8 expression for their survival. This came from experiments revealing the acute loss of a fraction of CD8 T cells following induced *Casp8* deletion specifically amongst mature CD8 T cells. In Casp8ΔT^CD2^ mice, such CASPASE8-dependent CD8 T cells may be rapidly purged as they are generated or first enter the periphery, such that the peripheral compartment becomes reduced in size in adult mice. The cells that remain, however, display normal life spans, even when transferred to a WT host environment. Kinase dead RIPK1 appeared to restore the naive CD8 compartment in Casp8ΔT^CD2^ mice, suggesting that the loss of some naive CD8 T cells was due to necroptosis. This is surprising because neither thymocytes nor naive T cells appear to express *Mlkl*, which is critical for induction of necroptosis [34]. These observations could be reconciled if *Mlkl* expression was induced transiently in some CD8 T cells, possibly during development or maturation, that could render them briefly susceptible to cell death in the absence of CASPASE8. If so, it would be easy to overlook or fail to detect *Mlkl* expression by a small transient population amongst the larger bulk of CD8 T cells in WT mice. Such a possibility is supported by the observations that type I interferons are reported to induce *Mlkl* in other cell types [35], and developing thymocytes do express an IFN-induced gene signature during development [36]. Whether such a mechanism is active in CD8 T cells remains to be determined, however.

We analysed mice with kinase dead RIPK1 to identify those phenotypes that were RIPK1 kinase dependent and resulting from induction of necroptosis in the absence of CASPASE8. Thymic cellularity and peripheral naive CD8 T cell numbers were rescued by RIPK1^D138N^ as was generation of CD8 CM and CD8 EM in both intact mice and in bone marrow chimeras. These findings are consistent with previous studies demonstrating that effector T cells are susceptible to necroptosis in the absence of CASPASE8, both following activation *in vitro* and during viral infection *in vivo* [17, 18, 33]. In contrast, CASPASE8-deficient CD8 VM T cells were only partially rescued by kinase dead RIPK1, while the profound loss of NKT cells was barely impacted at all by inactivation of RIPK1. It was also striking that CASPASE8-deficient T cells were highly resistant to cell death induction *in vitro*, in response to FAS or TNF stimuli, demonstrating that resting T cells are not obviously sensitized to necroptosis in the absence of CASPASE8, at least in response to TNFRSF stimuli. Further evidence of a necroptosis-independent CASPASE8-mediated survival mechanism came in the mixed bone marrow chimeras, where there were clear defects in the generation and/or persistence of CD4 CM and CD4 EM, which were not rescued by kinase dead RIPK1. Also, naive CD4 and naive CD8 T cells were both underrepresented in mixed bone marrow chimeras reconstituted with donors expressing RIPK1^D138N^, even though kinase dead RIPK1 rescued normal thymic development. In these cases, CASPASE8-deficient T cells were clearly at a disadvantage to WT cells, and this was not rescued by kinase dead RIPK1.

While CASPASE8 is a potent trigger of cell death, taken together, our findings suggest that another important role of CASPASE8 in T cells is to promote their survival. This is in part because of the important role CASPASE8 plays in repressing necroptosis, as was evident in developing thymocytes and in CD8 CM and CD8 EM. Indeed, when necroptosis was blocked in RIPK1^D138N^ mice, CASPASE8 deficiency resulted in a substantial expansion of CD8 EM compartment that resembles similar expansions observed in *Fas^lpr^* mice [37, 38] and likely represents a failure of FAS to kill CD8 EM in the absence of CASPASE8 and RIPK1 kinase activity, as also described in FADD and RIPK1-deficient mice [39]. However, there was also abundant evidence for a pro-survival function of CASPASE8 that was distinct from its role in repressing necroptosis. This function appears to be important in NKT cells, CD8 VM, CD4 CM, and CD4 EM and may also be required for optimal competitive fitness of both naive CD4 and naive CD8 T cells. The mechanism for this survival function remains obscure. We could not find any evidence that well-characterized survival signals that derive from TCR recognition of self-peptide MHC complexes or from IL-7R signalling were affected in the absence of CASPASE8. Oligomerization of CASPASE8 is associated with triggering apoptosis and active CASPASE8 is also responsible for targeted cleavage of RIPK1 [11, 12]. It is possible that CASPASE8 has other substrates whose cleavage is required for normal T cell survival. Alternatively, functions have been described for CASPASE8 that are independent of its proteolytic activity. Studies of TRAIL signalling in cell lines show that CASPASE8 has an adapter function during signalling that is required for NF-κB-dependent inflammatory signalling and induction of cytokine production downstream of TRAIL receptor, that does not require enzymatic activity by CASPASE8 [40]. Acute T cell survival is dependent upon tonic NF-κB signalling [30], and so it is possible CASPASE8 may be required for transmission of these signals. To explore these possibilities, future studies will need to address whether enzymatic or adapter function of CASPASE8 are required for RIPK1-independent survival mechanisms.

## Supplementary Material

kyaf016_Supplementary_Data

## Data Availability

Data is available upon reasonable request.

## Abbreviations

CBM: CARD11 BCL10 MALT1
CIAP: cellular inhibitor of apoptosis protein
IKK: inhibitor of kappa B kinase
IκB: inhibitors of kappa B
Nec1: necrostatin-1
RIPK1: receptor-interacting protein kinase 1
TAK1: transforming growth factor-beta activated kinase 1
TNFR1: tumour necrosis factor receptor 1
TRADD: tumour necrosis factor receptor 1–associated death domain protein
TRAF: tumour necrosis factor receptor–associated factor
