# Supplementary material for: Evidence for a RIPK1-independent survival mechanism for CASPASE-8 in αβ T cells
Source: Discov Immunol. 2025 Nov 28;4(1):kyaf016. doi: 10.1093/discim/kyaf016 (PMC12770974; doi:10.1093/discim/kyaf016)

Figure S1 - Limited Cre toxicity amongst CD8 EM and CD8 VM in CD8^{CreERT} mice. *CD8^{CreERT}* mice were treated with TAM for five consecutive days. At days 7 and 21, mice were culled and CD8 T cell compartment in lymph node and spleen enumerated. (A) Density plots show representative phenotypes in lymph nodes cells from TAM treated CD8^{CreERT} mice (n=4), untreated CD8^{CreERT} mice (n=5) and Cre -ve littermate controls (n=3) at day 21, and gates used to identify naive, EM, CM and VM subsets of CD8 T cells. (B) Scatter plots show total numbers of the indicated CD8 subset recovered from lymph node and spleen of the indicated strains and time points indicated after first TAM treatment. Cell numbers from Controls (Cre– and untreated CD8CreERT2) and TAM treated (d7 and d21) were compared by nonparametric unpaired Mann-Witney student's t test. ** < 0.01). Data are representative of 2 independent experiments.

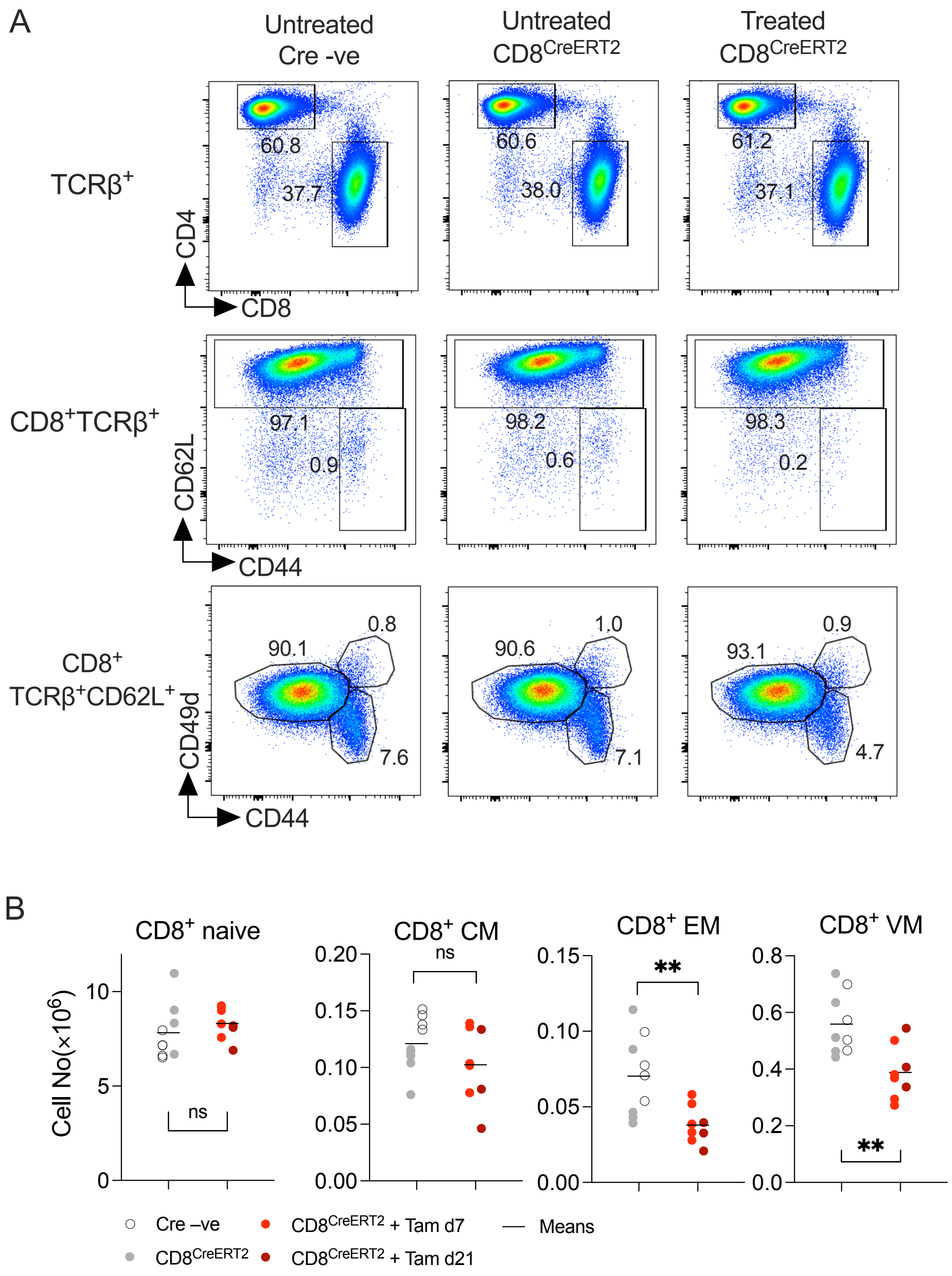

Figure S2 - Partial rescue of thymopoiesis in Casp8ΔT^{CD2} mice by kinase dead RIPK1. Thymus from Casp8ΔT^{CD2} RIPK1^{D138N} (n=23) and Cre –ve littermates (n=11) were enumerated analysed by flow. (A) Scatter plots show numbers of the indicated thymic subsets from Casp8ΔT^{CD2} RIPK1^{D138N} mice and Cre –ve littermates. (B) Density plots are of HSA vs CD1d-α-GalCer (CD1dTet) binding to total live thymocytes. CD4 vs CD8 expression is for CD1dTet^{+} cells. HSA vs CD44 expression is shown for CD4^{+} CD8$^{–}$ CD1dTet^{+} (CD4 NKT) cells and CD4$^{–}$CD8$^{–}$ CD1dTet^{+} (DN NKT) cells. Scatter plots summarise total numbers of CD4^{+} NKT and DN NKT recovered from the indicated strains.

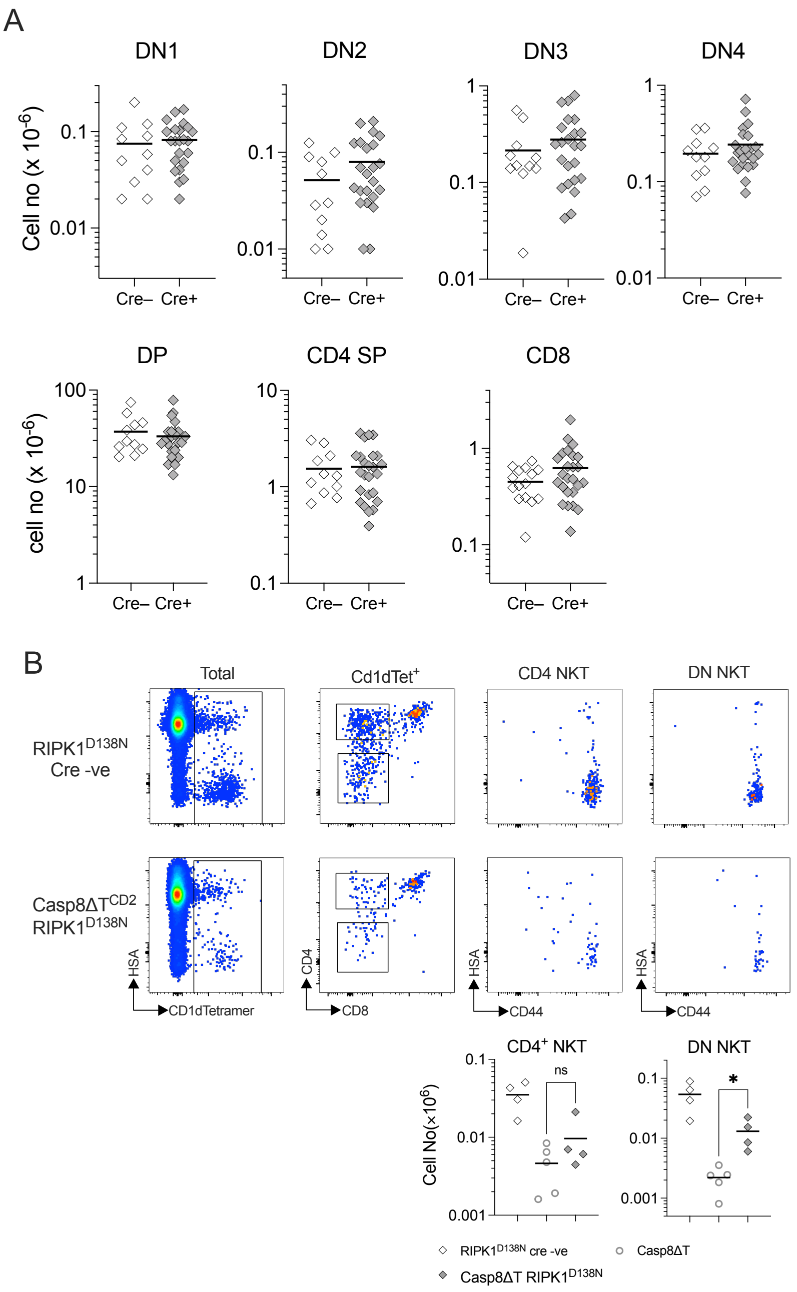

Figure S3 - Flow cytometry gating strategy to identify viable singlets.

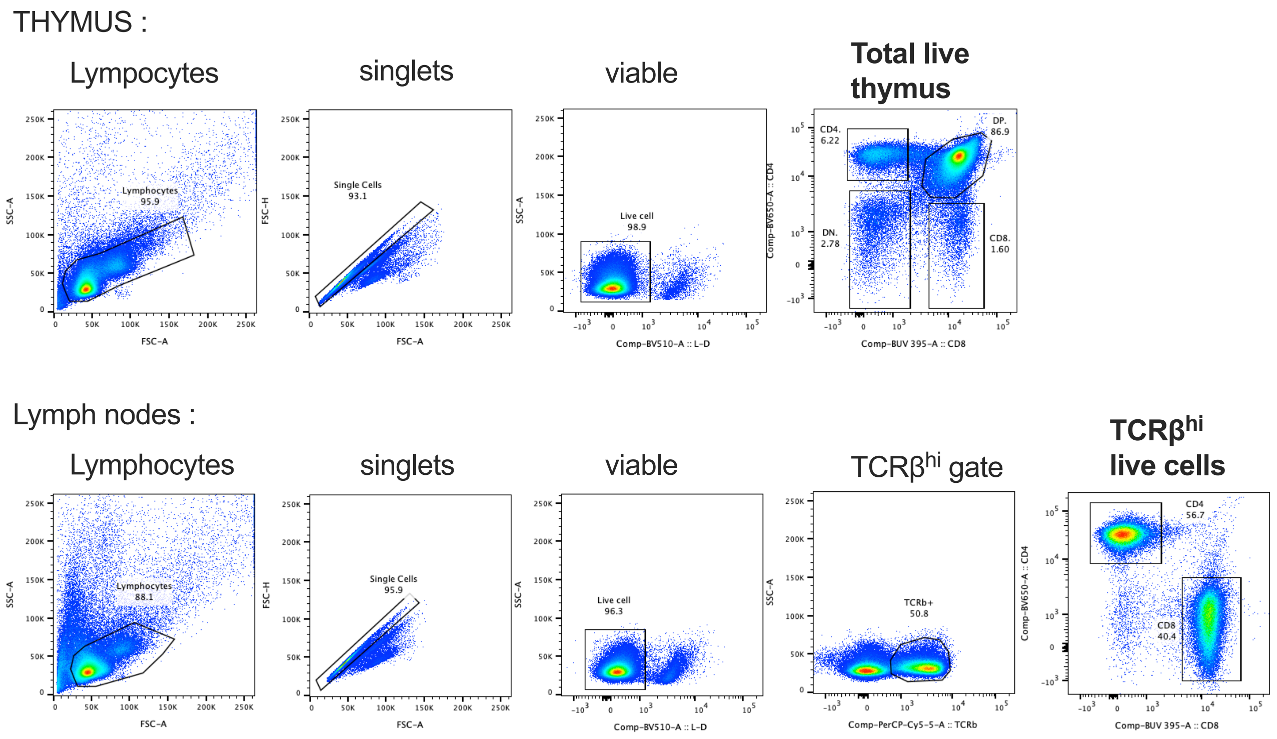

Supplement: kyaf016_Supplementary_Data [file kyaf016_supplementary_data.pdf]
